# Supplementary material for: Evolution of crop phenotypic spaces through domestication
Source: New Phytol. 2026 Feb 27;250(3):1948–63. doi: 10.1111/nph.71031 (PMC13062731; doi:10.1111/nph.71031)
Supplement: Supplementary file 1 — Fig. S1 Domestication syndrome in African rice. Fig. S2 Domestication syndrome in apple. Fig. S3 Domestication syndrome in cabbage. Fig. S4 Domestication syndrome in common bean. Fig. S5 Domestication syndrome in eggplant. Fig. S6 Domestication syndrome in einkorn wheat. Fig. S7 Domestication syndrome in grapevine. Fig. S8 Domestication syndrome in melon. Fig. S9 Domestication syndrome in maize. Fig. S10 Domestication syndrome in pearl millet. Fig. S11 Domestication syndrome in sugar beet. Fig. S12 Domestication syndrome in tomato. Fig. S13 Convergence in domestication‐associated traits. Fig. S14 Testing for convergence of domestication‐associated traits. Fig. S15 Correlation between the log ratio of domestic to wild NIRSleaf phenotypic space size with the ratio of domestic to wild genomic diversity. [file NPH-250-1948-s002.docx]

## *New Phytologist* Supporting Information

Article title: Evolution of crop phenotypic spaces through domestication

Authors: Arthur Wojcik, Harry Belcram, Agnès Rousselet, Manon Bouët, Marie Brault, Pierre Serin, Augustin Desprez, Renaud Rincent, Andreas Peil, Karine Henry, Cécile Marchal, Thierry Lacombe, Sylvain Glémin, Karine Alix, Pierre R. Gérard, Domenica Manicacci, Yves Vigouroux, Catherine Dogimont, Maud I. Tenaillon

Article acceptance date: 31 January 2026

The following Supporting Information is available for this article:

**Fig. S1** Domestication syndrome in African rice.

**Fig. S2** Domestication syndrome in apple

**Fig. S3** Domestication syndrome in cabbage

**Fig. S4** Domestication syndrome in common bean

**Fig. S5** Domestication syndrome in eggplant.

**Fig. S6** Domestication syndrome in einkorn wheat.

**Fig. S7** Domestication syndrome in grapevine.

**Fig. S8** Domestication syndrome in melon.

**Fig. S9** Domestication syndrome in maize.

**Fig. S10** Domestication syndrome in pearl millet.

**Fig. S11** Domestication syndrome in sugar beet.

**Fig. S12** Domestication syndrome in tomato.

**Fig. S13** Convergence in domestication-associated traits.

**Fig. S14** Testing for convergence in domestication-associated traits.

**Fig. S15** Correlation between the log ratio of domestic to wild NIRS_leaf_ phenotypic space size with the ratio of domestic to wild genomic diversity

**Table S1** Description of the 13 pairs of species with estimate of the domestication timing, domestication center used in this study, mating system and life cycle.

**Table S2** Passport data of sampled accessions.

**Table S3** Number of traits measured and growing conditions during phenotyping in each species.

**Table S4** List of measured traits for each species, and domestication-associated (DA) traits with corresponding q-values and percentage of DA-traits in each species.

**Table S5** Number of traits measured per species (columns) and number of species for which a given trait was measured (shared traits in rows). Traits are described Table S4.

**Table S6** Ratio of domestic (D) over wild (W) multivariate phenotypic space, mDPI, Pillai trace computed on all traits and on the control trait, and estimates from the literature of the ratio (D/W) of genomic diversity and Fst.

**Table S7** Average absolute pairwise correlation between traits, computed from all pairwise correlations in wild and domesticated forms.

**Fig. S1** Domestication syndrome in African rice. Spider-plot for quantitative traits with significance (*) between wild and domesticated forms at an FDR of 5% (a). Cleveland plot of qualitative traits (b). First two axes of the PCoA computed from NIRS_seed_ (c) and NIRS_leaf_ (d). In (a) the dots represent the mean values, with the shaded area showing the standard error. Abbreviation meaning of traits can be found in table S4.


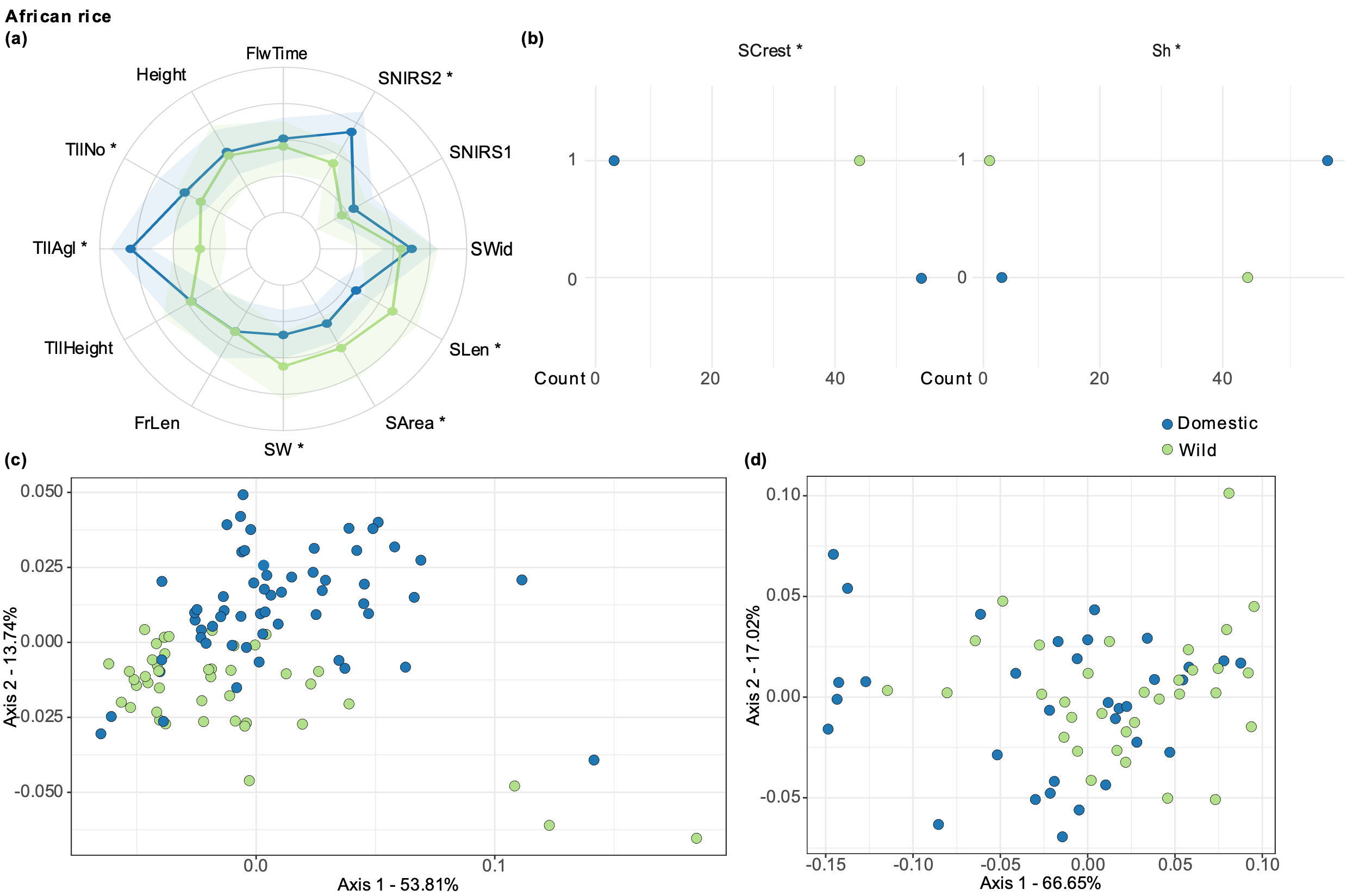
**Fig. S2** Domestication syndrome in apple. Spider-plot for quantitative traits with significance (*) between wild and domesticated forms at an FDR of 5% (a). First two axes of the PCoA computed from NIRS_leaf_ (b). In (a) the dots represent the mean values and the
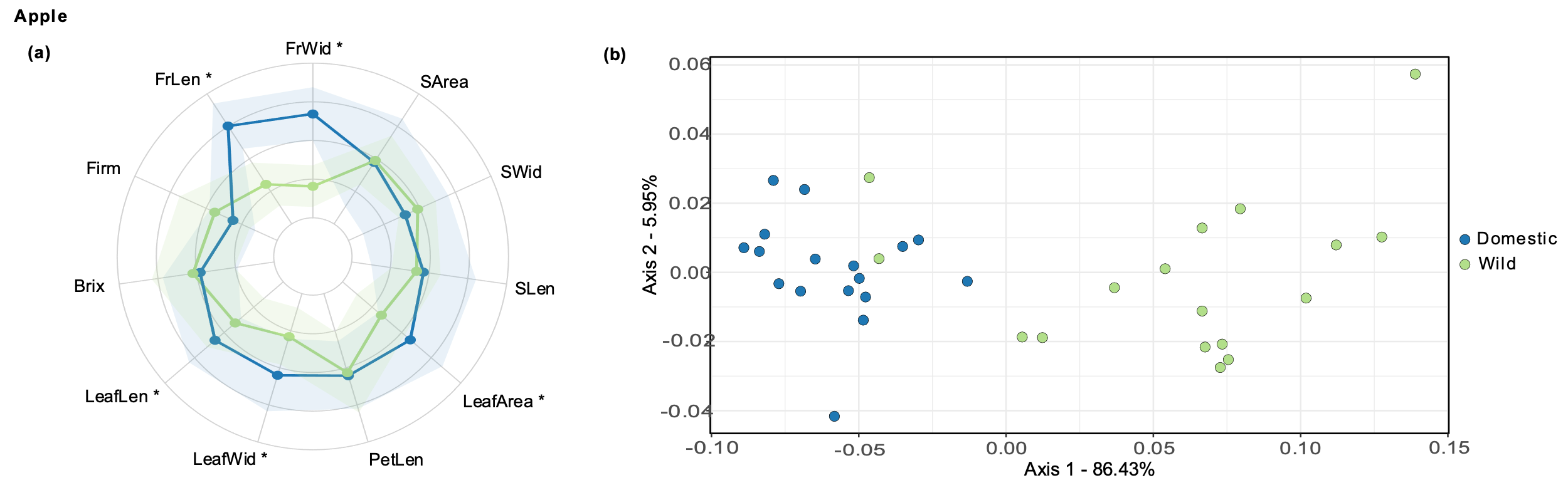
shaded areas denote standard error. Abbreviation meaning of traits can be found in table S4.

**
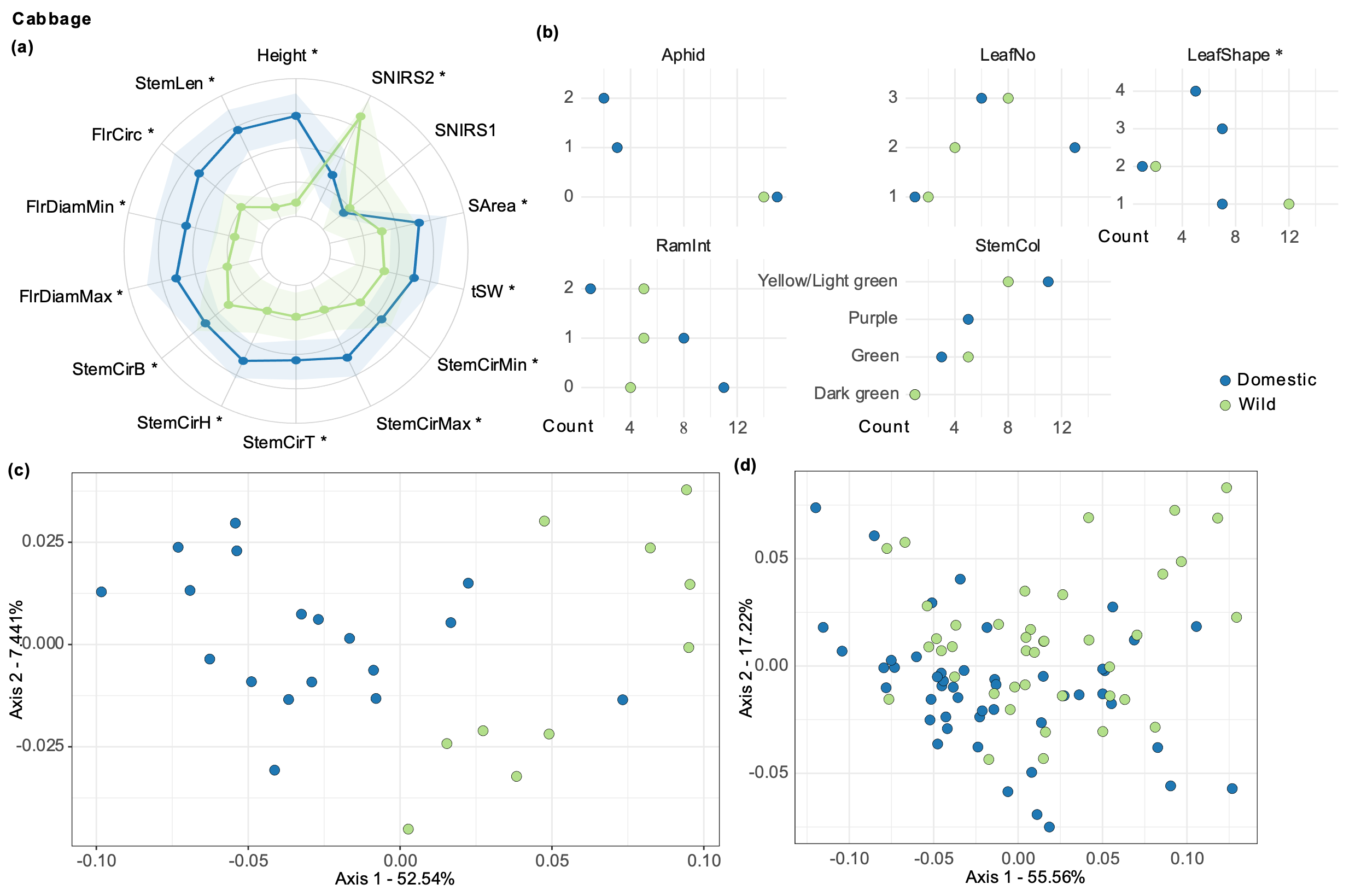
Fig. S3** Domestication syndrome in cabbage. Spider-plot for quantitative traits with significance (*) between wild and domesticated forms at an FDR of 5% (a). Cleveland plot of qualitative traits (b). First two axes of the PCoA computed from NIRS_seed_ (c) and NIRS_leaf_ (d). In (a), the dots represent the mean values and the shaded areas denote standard error. Abbreviation meaning of traits can be found in table S4.

**Fig. S4** Domestication syndrome in common bean. Spider-plot for quantitative traits with significance (*) between wild and domesticated forms at an FDR of 5% (a). Cleveland plot of qualitative traits (b). First two axes of the PCoA computed from NIRS_seed_ (c) and NIRS_leaf_ (d). In (a), the dots represent the mean values and the shaded areas denote standard error. Abbreviation meaning of traits can be found in table S4.


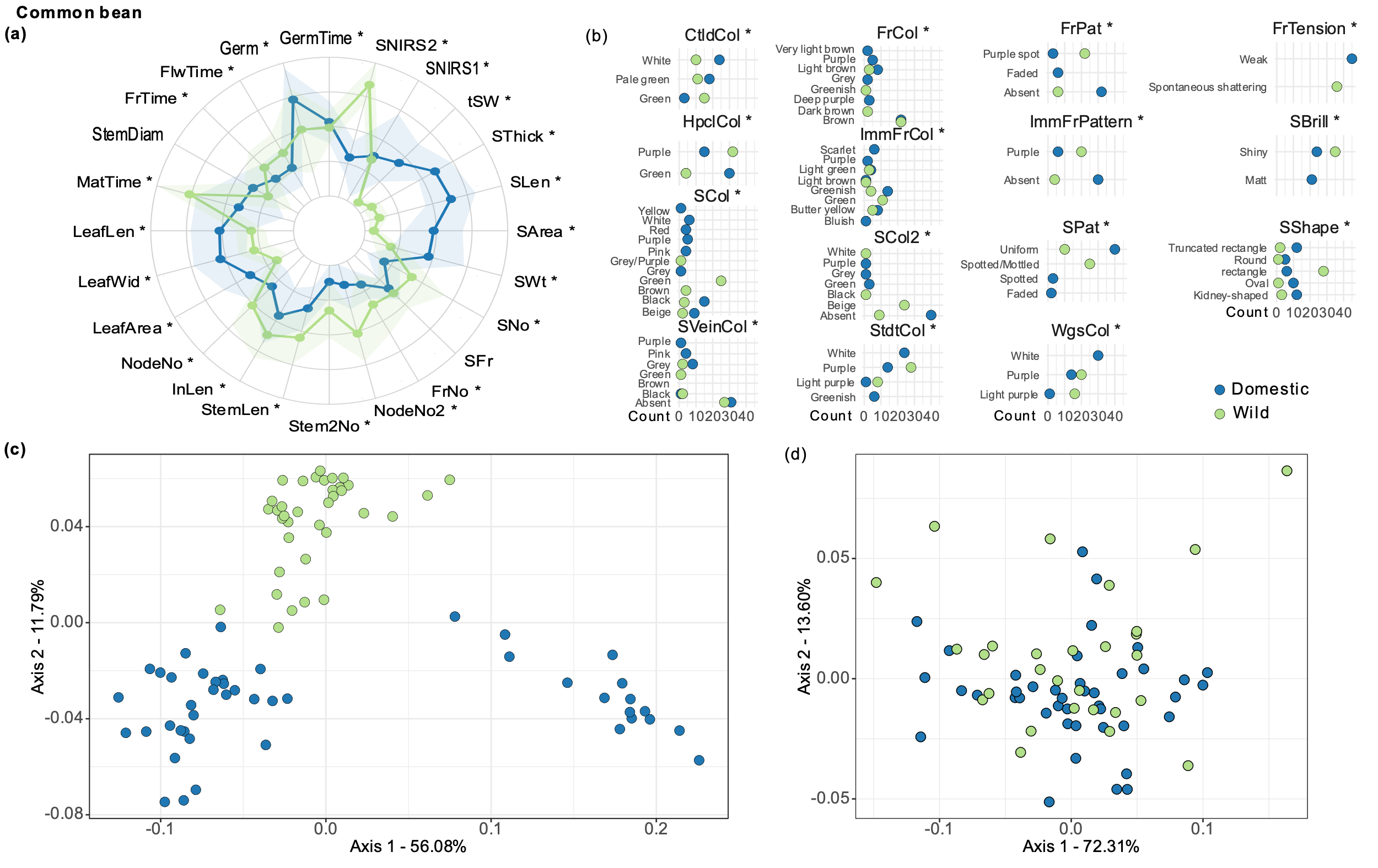


**Fig. S5** Domestication syndrome in eggplant. Spider-plot for quantitative traits with significance (*) between wild and domesticated forms at an FDR of 5% (a). Cleveland plot of qualitative traits (b). First two axes of the PCoA computed from NIRS_leaf_ (c). In (a), the dots represent the mean values and the shaded areas denote standard error. Abbreviation meaning of traits can be found in table **
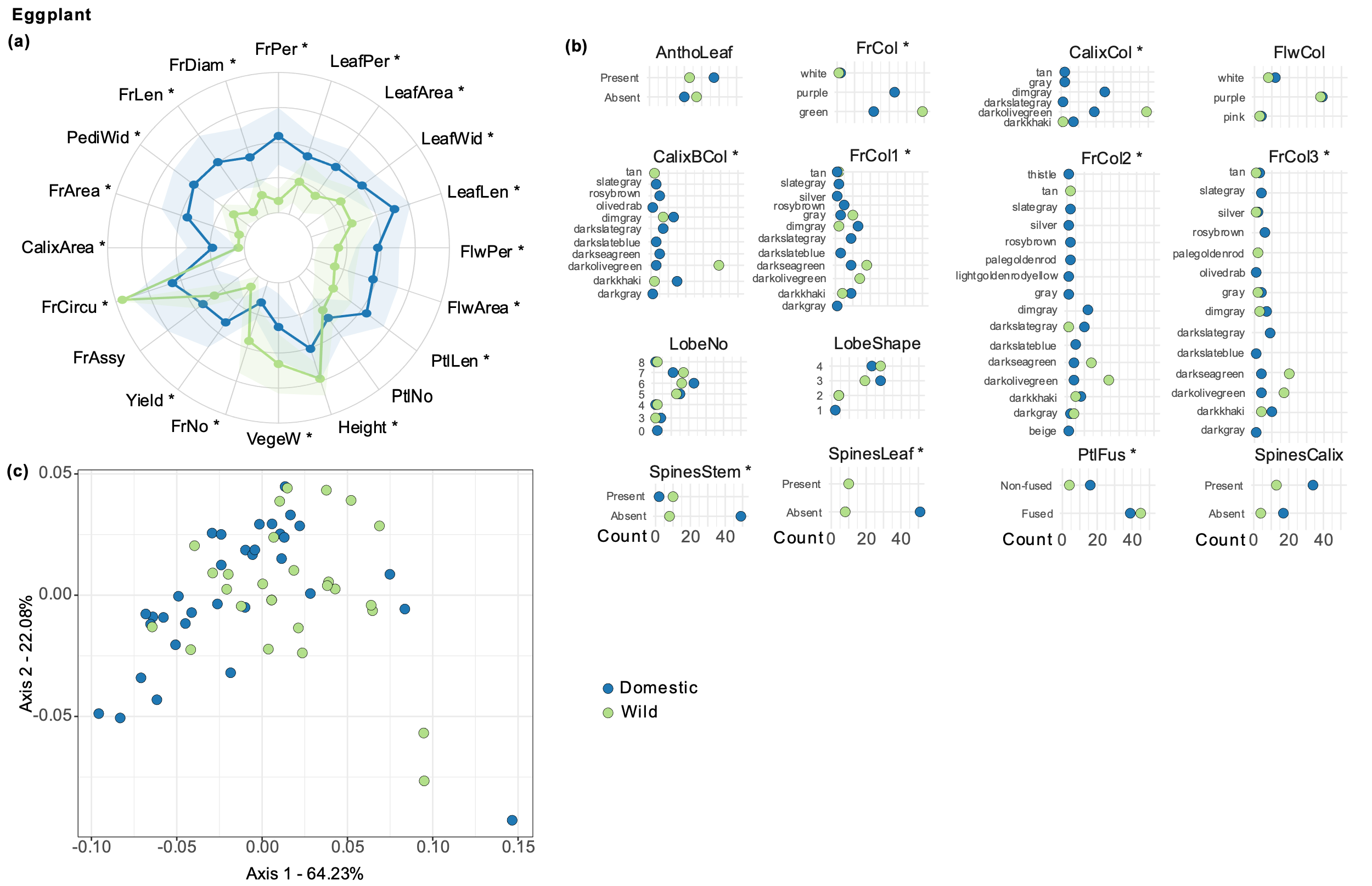
**S4.

**Fig. S6** Domestication syndrome in einkorn wheat. Spider-plot for quantitative traits with significance (*) between wild and domesticated forms at an FDR of 5% (a). First two axes of the PCoA computed from NIRS_seed_ (b) and NIRS_leaf_ (c). In (a), the dots represent the mean values and the shaded areas denote standard error. Abbreviation meaning of traits can be found in table S4.

**
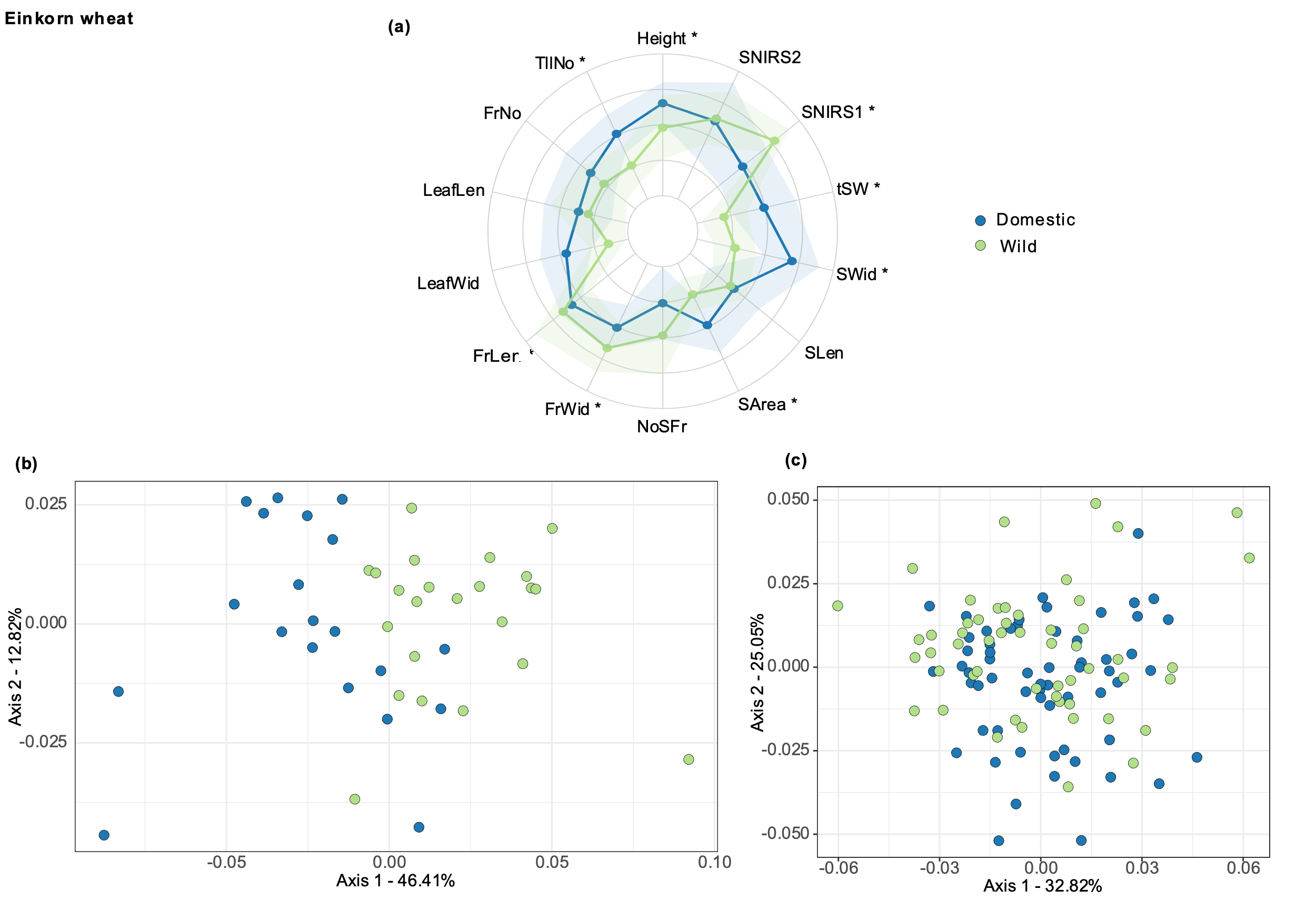
Fig. S7** Domestication syndrome in grapevine. Spider-plot for quantitative traits with significance (*) between wild and domesticated forms at an FDR of 5% (a). Cleveland plot of qualitative traits (b). First two axes of the PCoA computed from NIRS_leaf_ (c). In (a), the dots represent the mean values and the shaded areas denote standard error. Abbreviation meaning of traits can be found in table S4.

**
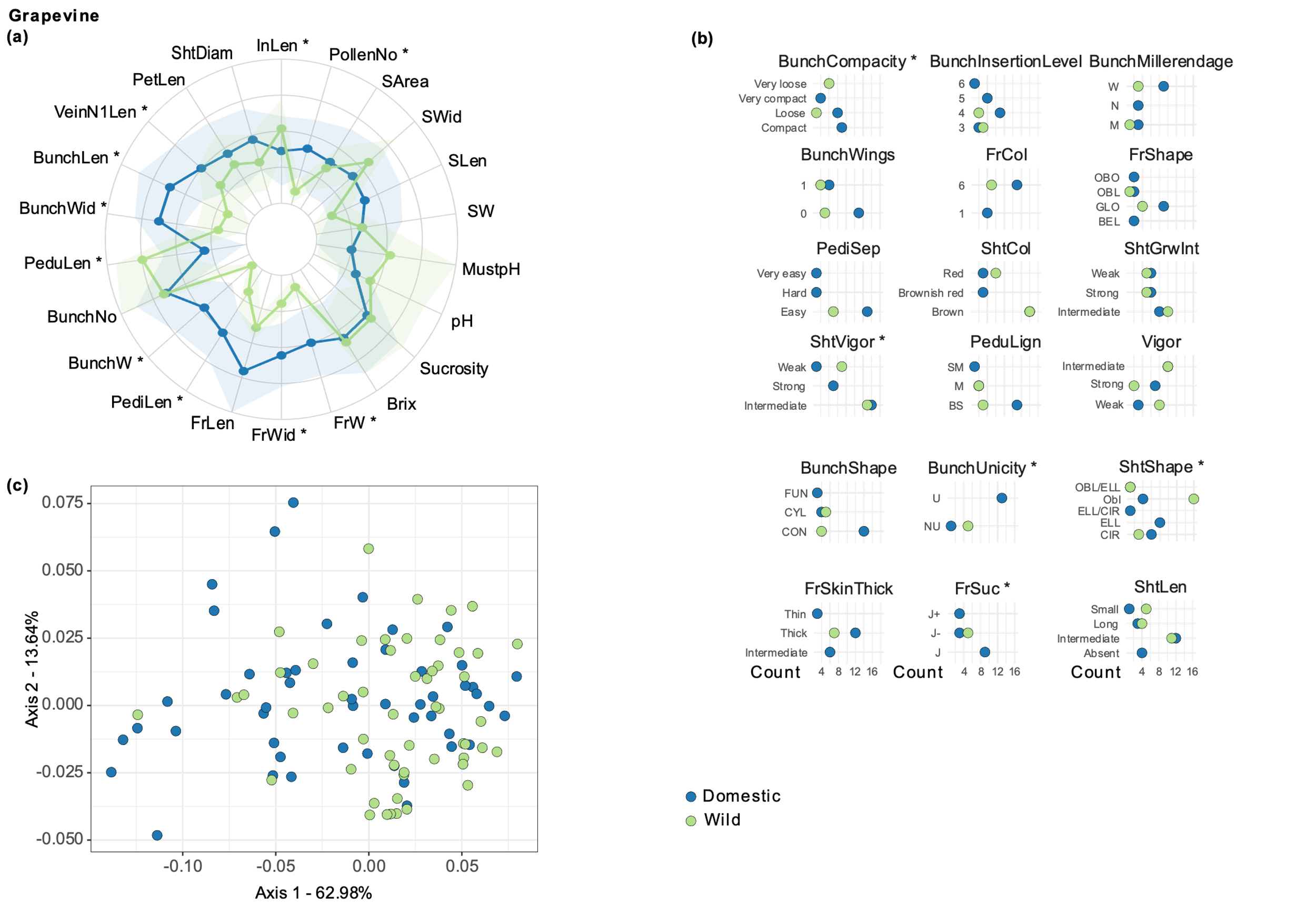
Fig. S8** Domestication syndrome in melon. Spider-plot for quantitative traits with significance (*) between wild and domesticated forms at an FDR of 5% (a). Cleveland plot of qualitative traits (b). First two axes of the PCoA computed from NIRS_seed_ (c) and NIRS_leaf_ (d). In (a), the dots represent the mean values and the shaded areas denote standard error. Abbreviation meaning of traits can be
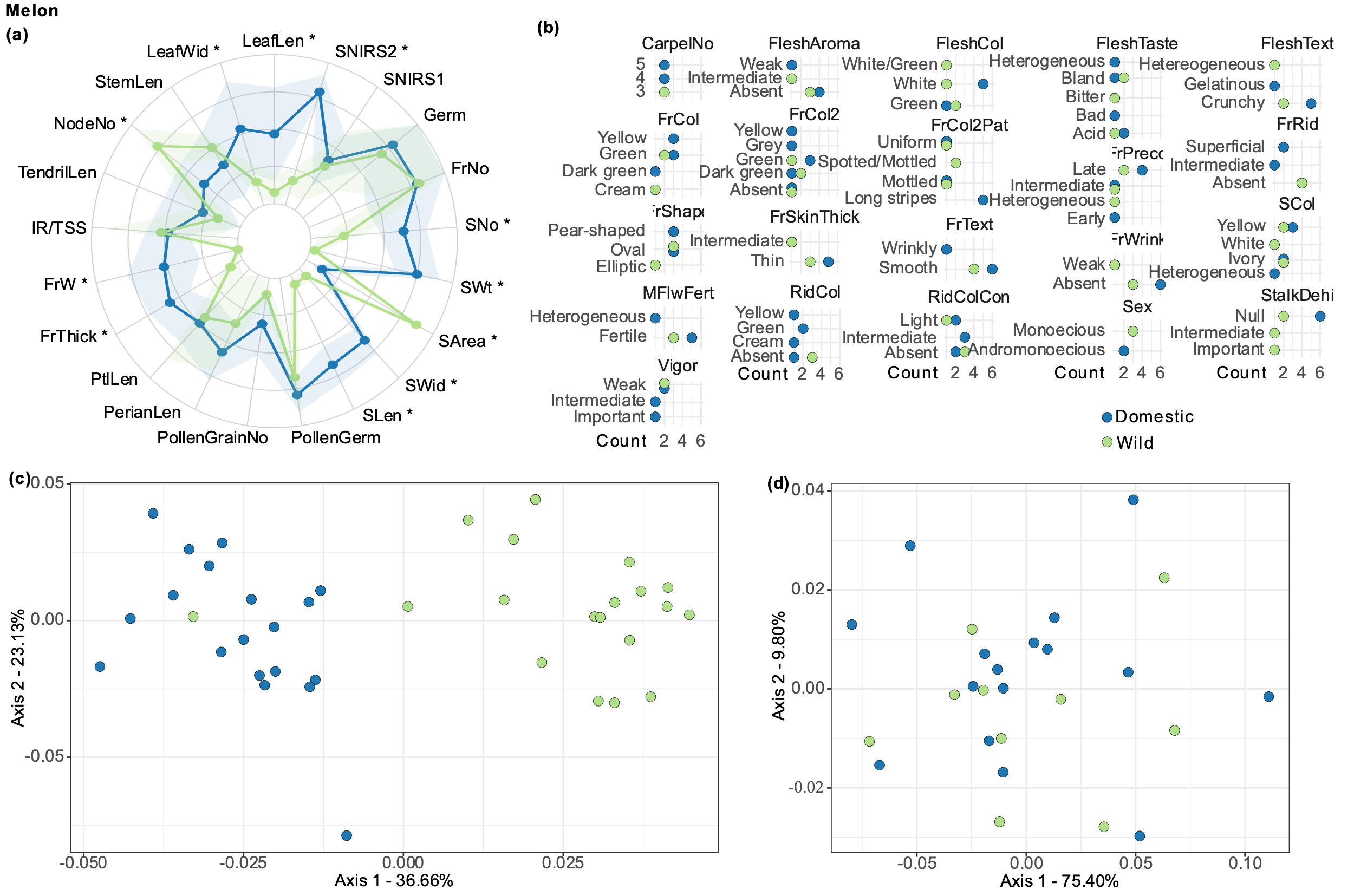
found in table S4.


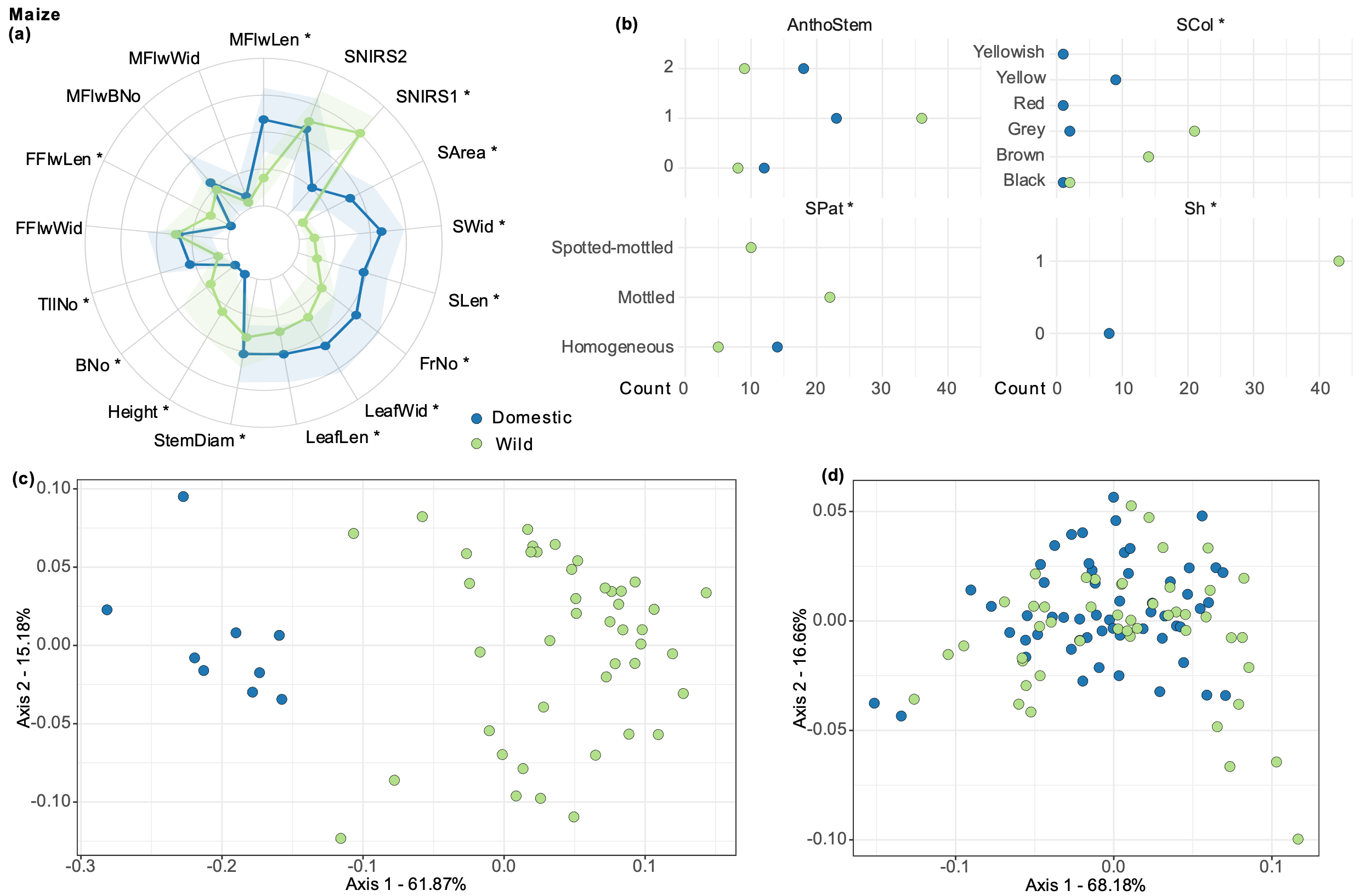
**Fig. S9** Domestication syndrome in maize. Spider-plot for quantitative traits with significance (*) between wild and domesticated forms at an FDR of 5% (a). Cleveland plot of qualitative traits (b). First two axes of the PCoA computed from NIRS_seed_ (c) and NIRS_leaf_ (d). In (a), the dots represent the mean values and the shaded areas denote standard error. Abbreviation meaning of traits can be found in table S4.

**
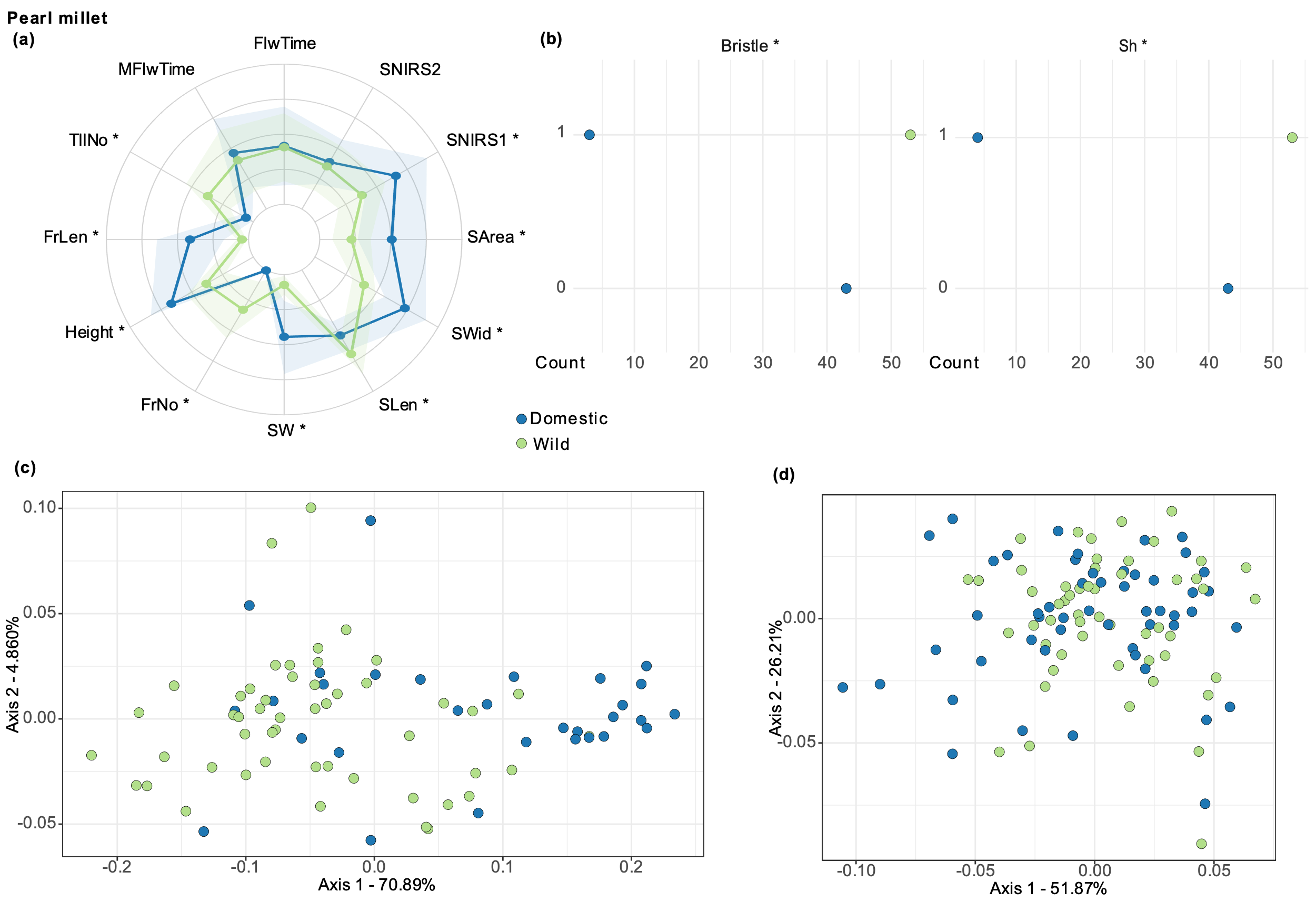
Fig. S10** Domestication syndrome in pearl millet. Spider-plot for quantitative traits with significance (*) between wild and domesticated forms at an FDR of 5% (a). Cleveland plot of qualitative traits (b). First two axes of the PCoA computed from NIRS_seed_ (c) and NIRS_leaf_ (d). In (a), the dots represent the mean values and the shaded areas denote standard error. Abbreviation meaning of traits can be found in table S4.


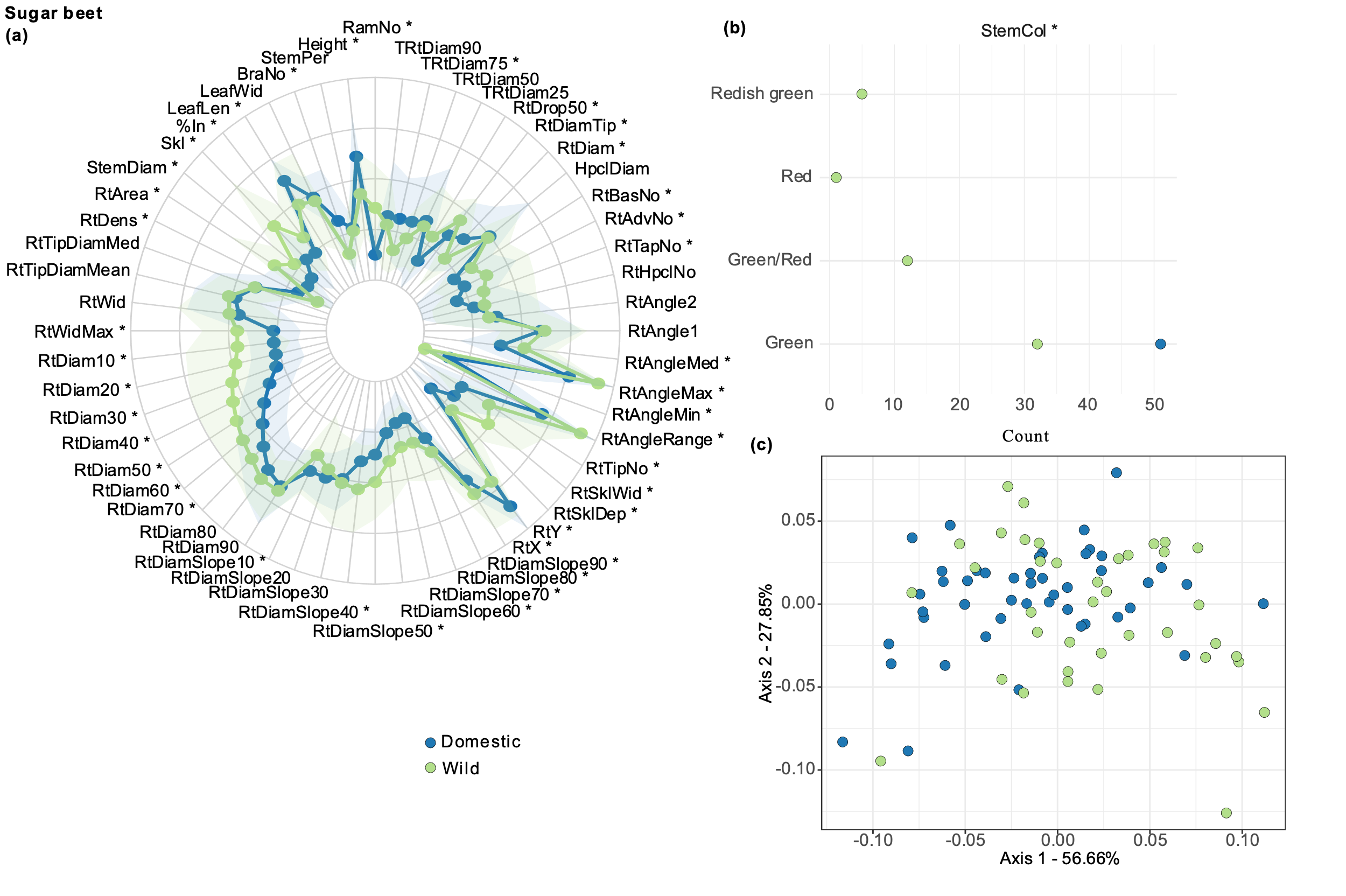
**Fig. S11** Domestication syndrome in sugar beet. Spider-plot for quantitative traits with significance (*) between wild and domesticated forms at an FDR of 5% (a). Cleveland plot of qualitative traits (b). First two axes of the PCoA computed from NIRS_leaf_ (c). In (a), the dots represent the mean values and the shaded areas denote standard error. Abbreviation meaning of traits can be found in table S4.

**
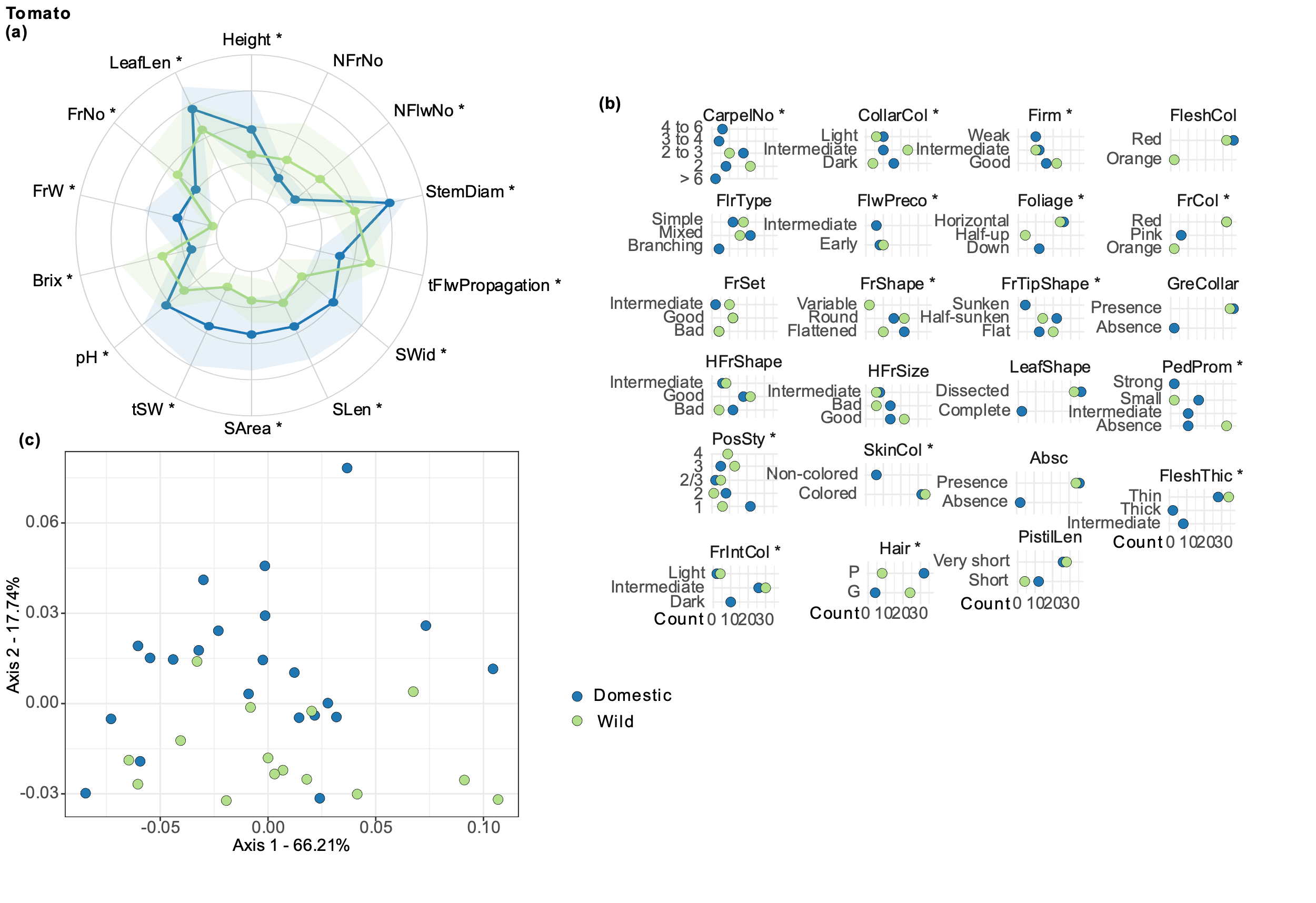
Fig. S12** Domestication syndrome in tomato. Spider-plot for quantitative traits with significance (*) between wild and domesticated forms at an FDR of 5% (a). Cleveland plot of qualitative traits (b). First two axes of the PCoA computed from NIRS_leaf_ (c). In (a), the dots represent the mean values and the shaded areas denote standard error. Abbreviation meaning of traits can be found in table S4.

**Fig. S13** Convergence in domestication-associated traits. The number of traits measured per species and shared between species is shown (a) along with the subset of domestication-associated traits (b).

**
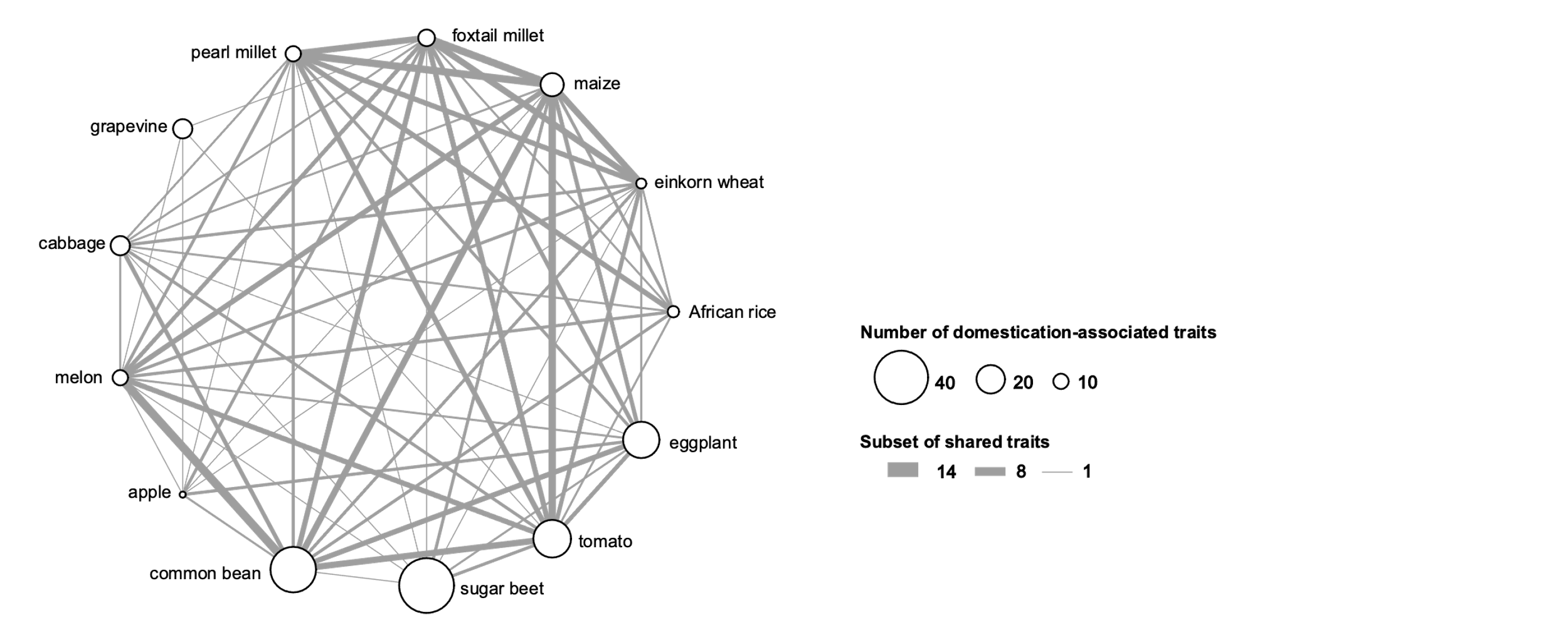
**

**
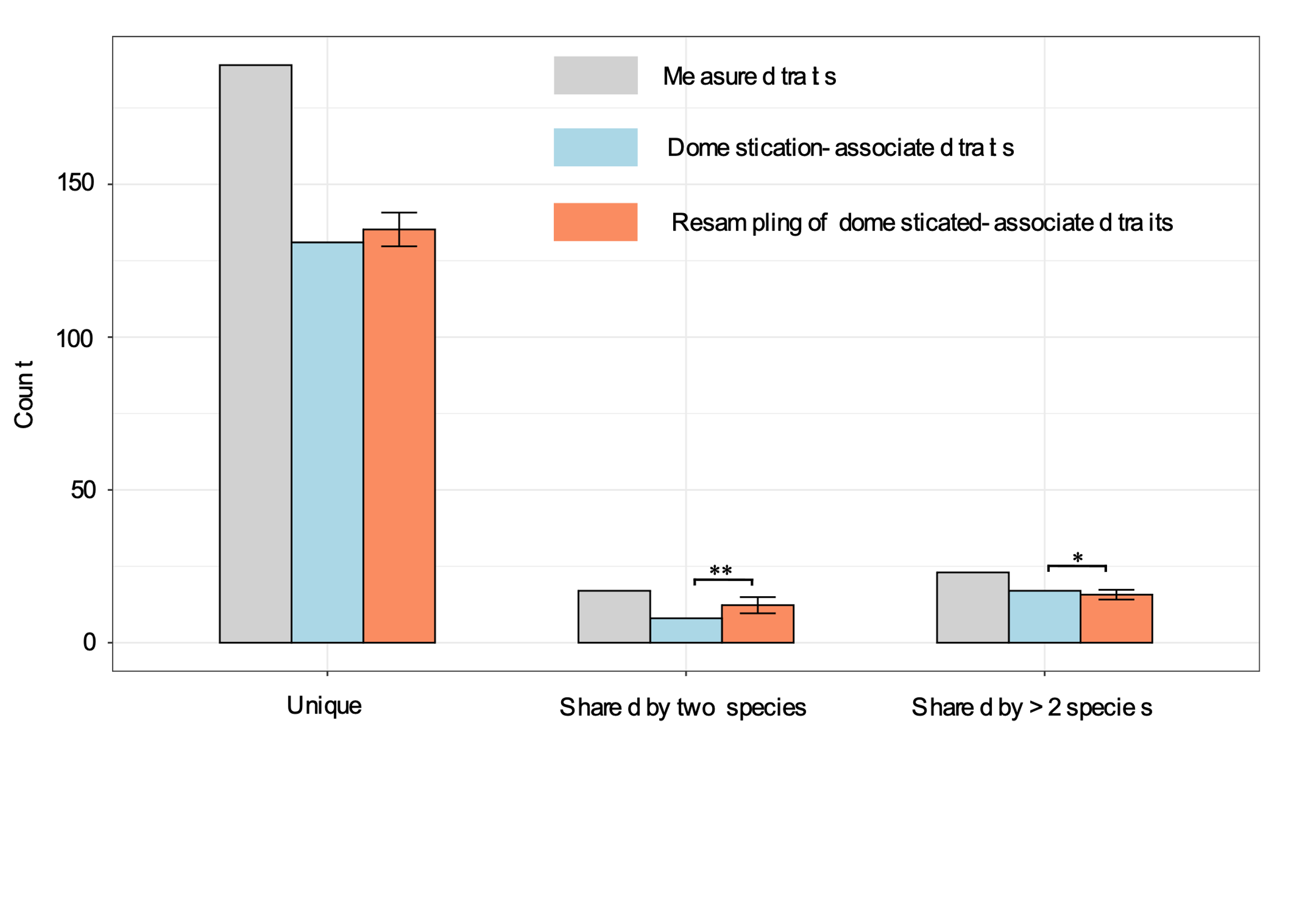
Fig. S14** Testing for convergence of domestication-associated traits. Observed number of domestication traits either unique or shared by two, or three or more species are shown with the expected number of shared domestication traits as established by a resampling procedure. Significance was tested by Student t-test (P-values are indicated when significant; * P < 0.05; ** P <0.01).

**Fig. S15** Correlation between the log ratio of domestic to wild NIRS_leaf_ phenotypic space size with the ratio of domestic to wild genomic diversity (values from the literature, Table S6).

**
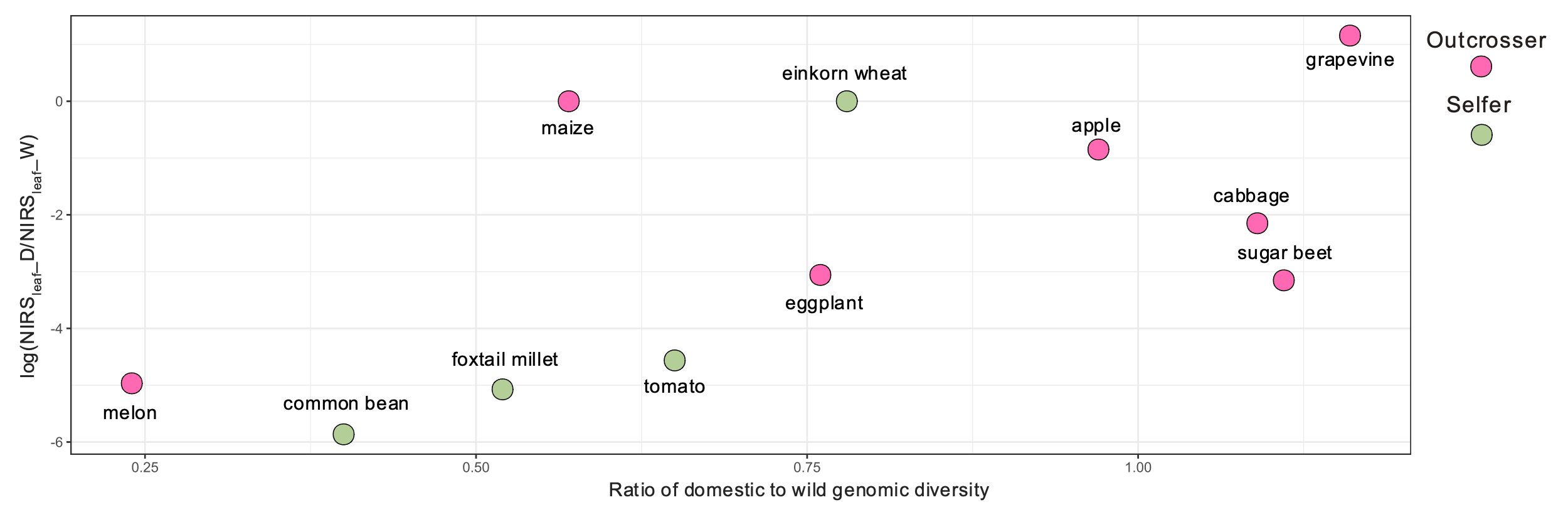
**

**Table S1** Description of the 13 pairs of species with estimate of the domestication timing, domestication center used in this study, mating system and life cycle.

**Table S2** Passport data of sampled accessions.

**Table S3** Number of traits measured and growing conditions during phenotyping in each species.

**Table S4** List of measured traits for each species, and domestication-associated (DA) traits with corresponding q-values and percentage of DA-traits in each species.

**Table S5** Number of traits measured per species (columns) and number of species for which a given trait was measured (shared traits in rows). Traits are described Table S4.

**Table S6** Ratio of domestic (D) over wild (W) multivariate phenotypic space, mPDI, Pillaicontrol, and estimates from the literature of the ratio (D/W) of genomic diversity and Fst.

**Table S7** Average absolute pairwise correlation between traits, computed from all pairwise correlations in wild and domesticated forms. P-values from Student t-tests.
